# Call order within vocal sequences of meerkats contains temporary contextual and individual information

**DOI:** 10.1186/s12915-020-00847-8

**Published:** 2020-09-09

**Authors:** Ramona Rauber, Bart Kranstauber, Marta B. Manser

**Affiliations:** 1 Animal Behaviour, Department of Evolutionary Biology and Environmental Studies, University of Zurich, Winterthurerstrasse, Zurich, Switzerland; 2 Kalahari Meerkat Project, Kuruman River Reserve, Van Zylsrus, Northern Cape South Africa; 3 Theoretical and Computational Ecology, Institute for Biodiversity and Ecosystem Dynamics, University of Amsterdam, Amsterdam, The Netherlands; 4 Mammal Research Institute, University of Pretoria, Pretoria, South Africa; 5 Center for the Interdisciplinary Study of Language Evolution (ISLE), University of Zurich, Zurich, Switzerland

**Keywords:** Animal vocal sequences, Combinatoriality, Sentinel behaviour, Call gradation, Individually distinct call patterns

## Abstract

**Background:**

The ability to recombine smaller units to produce infinite structures of higher-order phrases is unique to human language, yet evidence of animals to combine multiple acoustic units into meaningful combinations increases constantly. Despite increasing evidence for meaningful call combinations across contexts, little attention has been paid to the potential role of temporal variation of call type composition in longer vocal sequences in conveying information about subtle changes in the environment or individual differences. Here, we investigated the composition and information content of sentinel call sequences in meerkats (*Suricata suricatta*). While being on sentinel guard, a coordinated vigilance behaviour, meerkats produce long sequences composed of six distinct sentinel call types and alarm calls. We analysed recordings of sentinels to test if the order of the call types is graded and whether they contain additional group-, individual-, age- or sex-specific vocal signatures.

**Results:**

Our results confirmed that the six distinct types of sentinel calls in addition to alarm calls were produced in a highly graded way, likely referring to changes in the perceived predation risk. Transitions between call types one step up or down the a priory assumed gradation were over-represented, while transitions over two or three steps were significantly under-represented. Analysing sequence similarity within and between groups and individuals demonstrated that sequences composed of the most commonly emitted sentinel call types showed high within-individual consistency whereby adults and females had higher consistency scores than subadults and males respectively.

**Conclusions:**

We present a novel type of combinatoriality where the order of the call types contains temporary contextual information, and also relates to the identity of the caller. By combining different call types in a graded way over long periods, meerkats constantly convey meaningful information about subtle changes in the external environment, while at the same time the temporal pattern of the distinct call types contains stable information about caller identity. Our study demonstrates how complex animal call sequences can be described by simple rules, in this case gradation across acoustically distinct, but functionally related call types, combined with individual-specific call patterns.

## Background

The combinatorial diversity seen in the reuse and recombination of a finite set of smaller meaningless acoustic elements into meaningful units, which are then combined into infinite structures of higher-order phrases, is unique to human language [1, 2]. Yet, producing long sequences composed of smaller units has also been demonstrated across non-human animals, where producers combine multiple calls into larger meaningful structures. Some animal vocal sequences, in particular songs produced by birds and marine mammals, consist of smaller meaningless vocal units, which are not produced by themselves and thus can be considered to have no function but are combined into a meaningful overall sequence [3]. In contrast, when animals produce a series of meaningful units, they typically only combine two different call types, resulting in a meaning related to the meaning of its parts or in a new meaning different from the meaning of each call type separately (reviewed in [4]).

Information is not only conveyed by the composition of distinct call types into sequences, but also the temporal structures of repeated sound elements within a larger sequence can contain meaningful information [4]. In particular, information about predatory threats and an individual’s related level of arousal has been demonstrated to be reflected by changes in the number of repeated elements or the inter-element intervals [5, 6]. A few primate species have furthermore been described to combine distinct call types into larger sequences, where the proportional distribution of calls and transitional probabilities among call types contain contextual information [7, 8]. However, the precise mechanisms underlying how the information in these large sequences is conveyed are often less clear.

Although an increasing body of data shows that animals apply diverse combinatorial mechanisms to combine single calls into larger structures, there still is a gap between songs (which typically contain information about caller attributes) and call combinations (which contain contextual information)—with no intermediate forms described so far. Context and information content of these different structures vary across taxa (reviewed in [9]). In some avian species including marsh warblers (*Acrocephalus palustris*) and zebra finches (*Teaniopygia palustris*), the sequences are used to advertise male quality through the variation in complexity of the produced sequences [10, 11]. Sequences can contain identity information, both on an individual level [12, 13] and on a group level or local scale, i.e. neighbours versus strangers [14]. In contrast, call combinations have been demonstrated to contain meaningful, context-specific information based on the temporal ordering of the units contained in longer sequences [3, 7]. For example, bonobos (*Pan paniscus*) combine five acoustically graded call types into longer, mixed sequences containing information about the type of food encountered [8]. Despite the increasing evidence for meaningful vocal sequences across contexts, little attention has been paid to individual differences in the structure and composition of animal vocal sequences and the potential to contain further information about group identity, caller identity, age or sex.

In this study, we applied recently introduced analytical methods, which allow to systematically characterise and analyse call sequences of animal vocalisations (reviewed in [9, 15]) to investigate the combinatorial features and information content of a structurally complex call sequence produced by meerkats. Meerkats have been demonstrated to frequently combine calls across both social and predatory contexts [16]. They are small, highly social mongoose living in cooperatively breeding groups from three up to 50 (average group size 17) individuals [17] mostly occurring in savanna and semi-arid habitats in southern Africa. When foraging between vegetation and while digging for prey in the sand, meerkats have a limited view of their surroundings, making them rely heavily on acoustic rather than visual communication. Meerkats frequently show sentinel behaviour where one individual climbs on an elevated position and scans the area for the presence of predators [18]. If a sentinel spots a predator, they produce functionally referential alarm calls containing information about the type of predator (i.e. terrestrial or aerial) as well as graded information about the urgency level [19, 20], allowing foraging group members to adjust their escape behaviours accordingly [21].

**Fig. 1:**
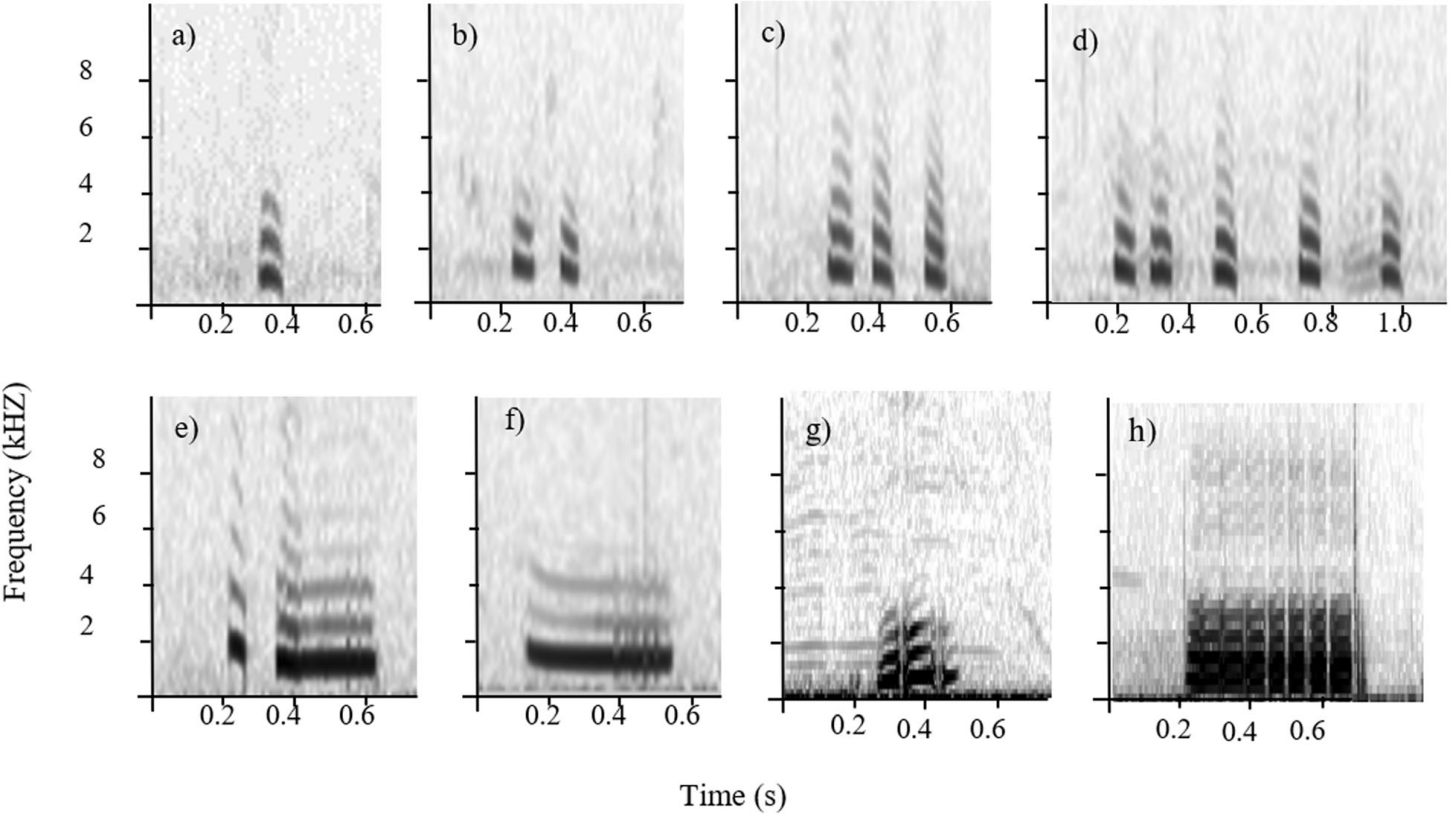
Spectrograms of the six sentinel call types **a** single-note call (sn), **b** double-note call (dn), **c** triple-note call (tn), **d** multiple-note call (mn), **e** di-drrr call and **f** wheek call and two examples of alarm calls: **g** medium-urgency terrestrial alarm and **h** high-urgency terrestrial alarm. Sentinel short-note calls: **a**–**d**, sentinel calming calls: **a** and **b** and sentinel warning calls: **e** and **f**

Besides alarm calls, meerkat sentinels continuously produce a series of calls composed of six discrete sentinel call types including single-note, double-note, triple-note, multiple-note, di-drrr and wheek calls (Fig. 1) [22]. Previous work on the function of different sentinel calls demonstrated that single and double-note calls act as ‘all-clear’ signal to the rest of the group and have since been referred to as sentinel calming calls [23]. In contrast, sentinel warning calls, including di-drrr and wheek calls, function as a pre-stage of alarm calls, often given during situations of increased perceived risk [23]. Besides the information about the perceived predation risk, foraging group members adjust their own vigilance behaviour in response to playbacks of sentinel calls depending on the identity and experience of the caller [24], highlighting the importance of individual distinctiveness in these calls and/or potentially the whole sequence. Despite understanding the functions of some of the six sentinel calls and that all meerkats produce all six described sentinel call types from the moment they first show guarding behaviour (Rauber & Manser, in prep.), the composition of the produced sentinel sequences—including order and potential gradation of the call types—as well as whether these sequences contain individual- or group-specific signatures remains unknown.

Here we investigated the combinatorial structure and the order of the different call types produced within sentinel sequences. Based on previous playback experiments manipulating the perceived risk of sentinels [23] and the acoustic properties of the six sentinel call types [22], we expected that the number of calls in the short-note calls, i.e. single-note (sn), double-note (dn), triple-note (tn) and multiple-note (mn) calls (Fig. 1), contains information about the perceived risk and thus should be given in a graded way. Based on the function and the acoustic structure of the warning sentinel call types (di-drrr and wheek calls; Fig. 1), we expected the order to be di-drrr, wheek and then alarm calls related to increased risk. Thus, we predicted an overall gradation from the single-note call type, to the double-note, triple-note, multiple-note, didrr, wheek and finally changing into alarm calls. Furthermore, we tested if there were consistent current group-, natal group-, individual-, age- or sex-specific calling patterns allowing receivers to gain additional information about the caller. If the call sequences were mainly reflecting the social or ecological environment signallers perceive, we would expect group- or natal group-specific signatures to emerge as a result of groups sharing the same environment. However, if it is mainly the information about signaller identity or characteristics, which may allow group members to adjust their responsiveness to the sentinel sequences, then we would expect not group signatures but individual-, age- or sex-specific signatures to evolve.

## Results

**Fig. 2:**
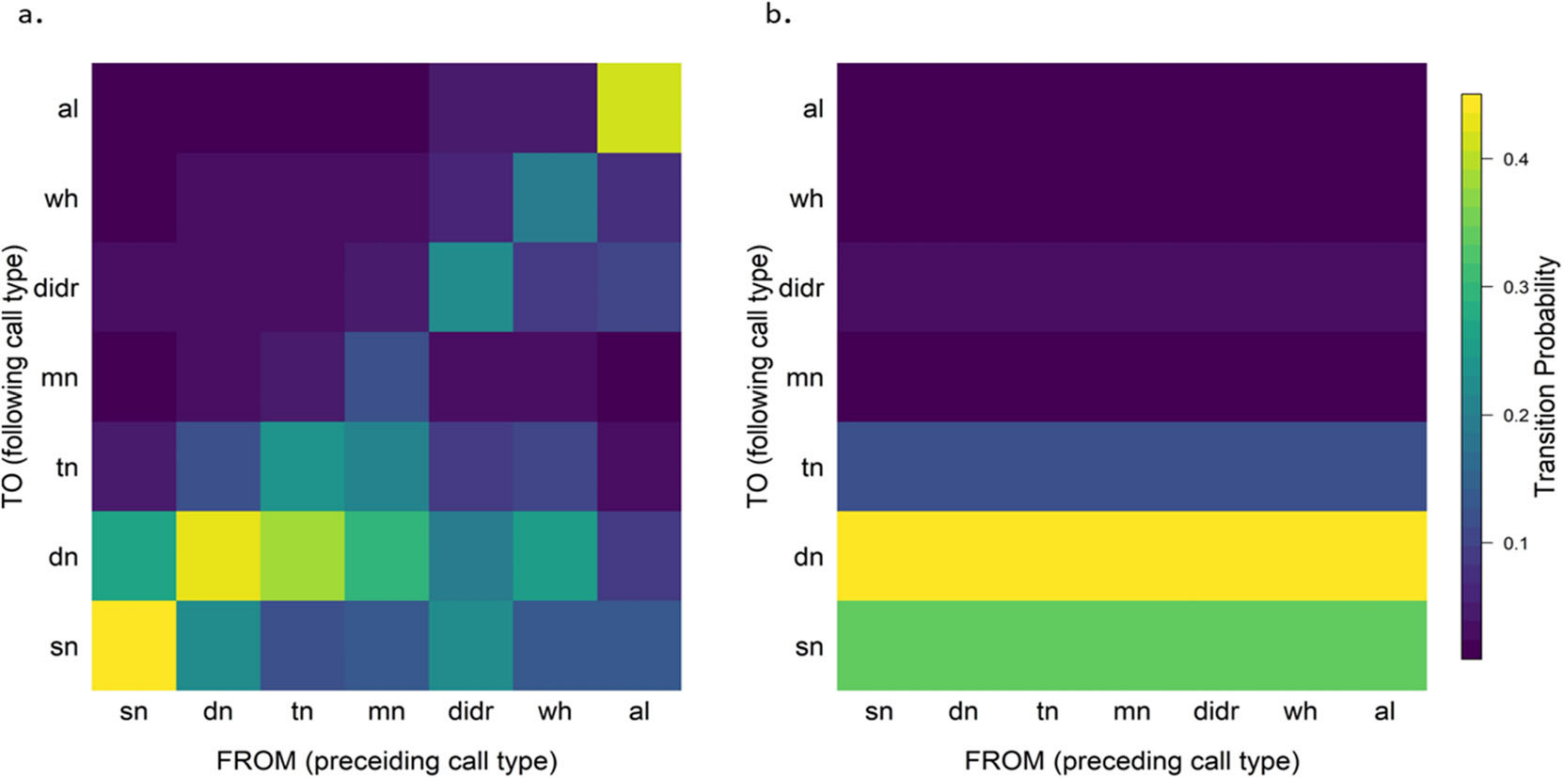
**a** Observed transition probabilities between all the six sentinel call types and alarm calls. **b** Expected transition probabilities for all six sentinel call types and alarm calls based on the frequency of each call type (i.e. if calls were given in a random order)

Randomising the recorded call sequences and comparing them to observed transition sequences (Fig. 2) showed that repetitions of the same call type were highly over-represented, i.e. observed number of transitions were above the expected confidence interval (CI), while all other transitions were under-represented, i.e. observed number of transitions were below the expected CI (Table 1). This means that independent of which call type was produced by the sentinel, the following call type would most likely be the same as the previous call type. Focusing on transitions between call types (by keeping the number of repetitions of the same call type constant, while randomising the transitions between call types), resulted in a highly graded pattern. We found that transitions that diverted one level from the zero diagonal (i.e. the self-repetitions; see Additional file: Fig. S1) were over-represented, while both the second and third diagonals were under-represented (Table 2). The under-representation of the second and third diagonals indicates that skipping a step in the gradation and going from a triple-note call either up to a didrr call or down to a single-note call happened significantly less than what was expected by chance (Table 2). The resulting overall gradation in order of increasing perceived risk was single-note, double-note, triple-note, multiple-note, didrr, wheek and finally alarm calls.

**Table 1:** Comparison between the number of transitions expected for each diagonal (95% CI) from the randomised sequences when all call transitions were randomised (null model) and the observed sequences

Diagonal	95%CI no. transitions (null)	Observed no. transitions	Interpretation
−6 (6 levels up)	129–160.5	75	Under
−5 (5 levels up)	233–280.1	128	Under
−4 (4 levels up)	487.4–542	441	Under
−3 (3 levels up)	482.5–540	386	Under
−2 (2 levels up)	895.9–995.7	789	Under
−1 (1 level up)	5120.9–5296.2	4849	Under
0 (repetition)	11,873.5–12,103	13,049	Over
1 (1 level down)	5105.9–5296.7	4834	Under
2 (2 levels down)	905–992.7	739	Under
3 (3 levels down)	493.4–550.6	403	Under
4 (4 levels down)	486.5–540.1	446	Under
5 (5 levels down)	239.5–281.6	156	Under
6 (6 levels down)	130–159	81	Under

**Table 2:** Comparison between the number of transitions expected for each diagonal (95% CI) from the randomised sequences when replications (zero diagonal) were kept constant (in order to focus on transitions between calls; null model) and the observed sequences

Diagonal	95%CI no. transitions (null)	Observed no. transitions	Interpretation
−6 (6 levels up)	70–94	75	As expected
−5 (5 levels up)	159–194	128	Under
−4 (4 levels up)	424–477	441	As expected
−3 (3 levels up)	424.9–490	386	Under
−2 (2 levels up)	791.9–873.0	789	Under
−1 (1 level up)	4612.9–4730.1	4849	Over
0 (repetition)	13,049–13,049	13,049	Kept constant
1 (1 level down)	4609.9–4730	4834	Over
2 (2 levels down)	784.9–870	739	Under
3 (3 levels down)	422–484	403	Under
4 (4 levels down)	420.9–473	446	As expected
5 (5 levels down)	154–193	156	As expected
6 (6 levels down)	69–92	81	As expected

**Fig. 3:**
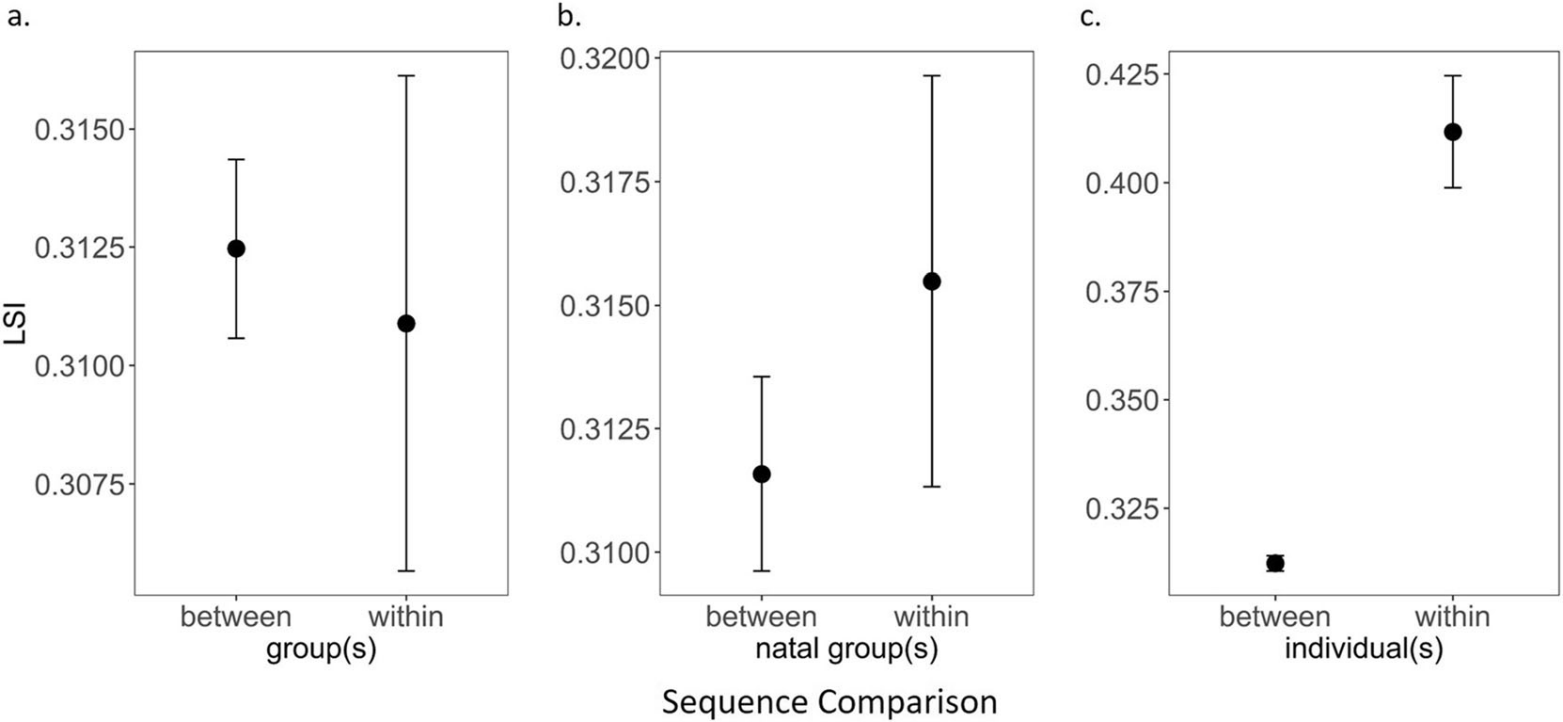
Sequence similarity (Levenshtein Similarity Index LSI) of sentinel sequences recorded from **a** adult individuals from the same or different groups, **b** adult individuals from the same or different natal groups and **c** within and between adult individuals

Investigating the variation in sentinel sequence composition in adult meerkats resulted in high sequence similarity (i.e. Levenshtein Similarity Index (LSI)) within bouts as well as within individuals. Comparison of the first and last third of sequences produced by adults resulted in similarity scores of 0.44 ± 0.19, indicating high consistency within a sentinel period. Regarding the presence of group or natal group signature in sentinel sequences, there was no indication that individuals within groups (Mann-Whitney *U* = 58,074, adjusted *p* = 1; Fig. 3a) or from identical natal groups (Mann-Whitney *U* = 58,074, adjusted *p* = 1; Fig. 3b) showed any shared patterns. In contrast, we found that sentinel sequences were more consistent, i.e. higher sequence similarity scores, within compared to between individuals (Mann-Whitney *U* = 3,953,700, *N* = 60 individuals, *n* = 162 sequences, adjusted *p* < 0.001; Fig. 3c) indicating individually distinct calling patterns. In particular, we found that the sequences composed of short-note calls (single-, double-, triple- and multiple-note calls) were significantly more similar within individuals compared to between individuals (Mann-Whitney *U* = 6,659,600, *N* = 60 individuals, *n* = 162 sequences, mean ± sd within ID = 0.43 ± 0.14; between ID = 0.35 ± 0.14, adjusted *p* < 0.001). However, when analysing only sequences consisting of sentinel warning calls and alarm calls (by artificially replacing any other calls in between with a single X), there was no difference between within-individual and between-individual sequence similarity (Mann-Whitney *U* = 58,074, *N* = 60 individuals, *n* = 162 sequences, mean ± sd within ID = 0.23 ± 0.14; between ID = 0.22 ± 0.13, adjusted *p* = 1).

**Fig. 4:**
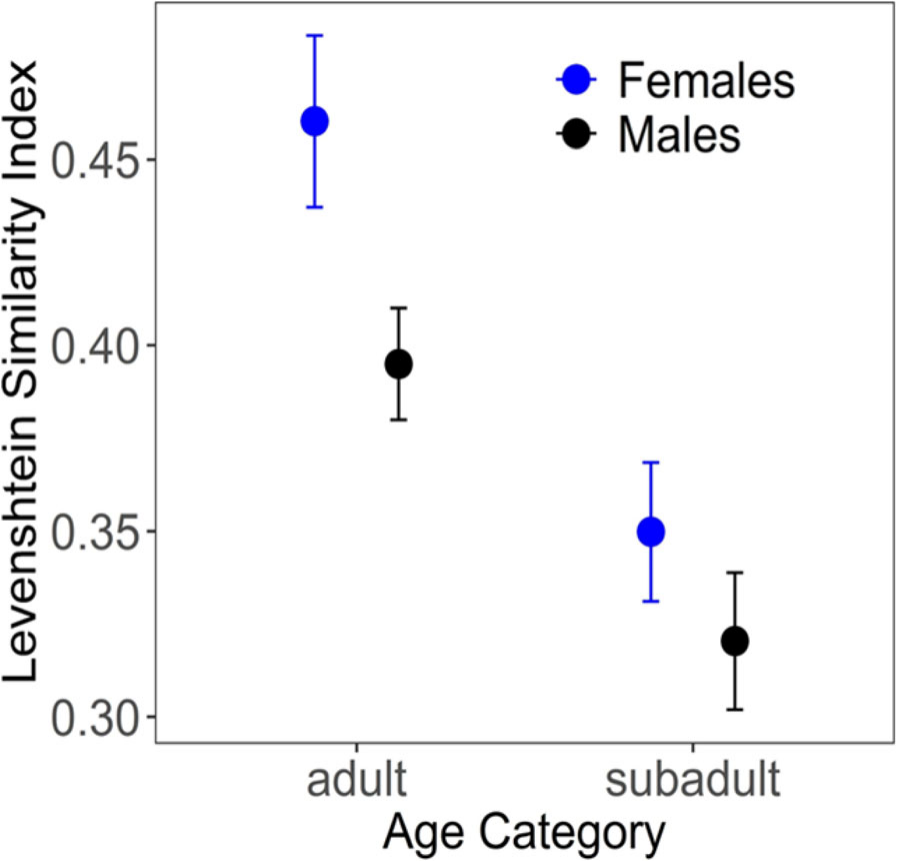
Within-individual sequence similarity (Levenshtein Similarity Index LSI) of sentinel sequences recorded from female (blue) and male (black) adults and subadults

Call sequences produced by subadults showed similar consistency scores within periods as found for sentinel sequences produced by adults, indicating high sequence consistency within a sentinel period (mean LSI ± sd = 0.4 ± 0.21). In contrast, within individuals, sequence similarity scores were significantly lower for subadults than for adults, indicating larger variation between recordings of the same individual in subadults (Mann-Whitney *U* = 158,810, *N* = 39 individuals, *n* = 129 sequences, adjusted *p* < 0.001, Fig. 4). Accordingly, for subadults there was no significant difference in consistency when comparing recordings from within compared to between individuals (Mann-Whitney *U* = 22,885,000, *N* = 39 individuals, *n* = 129 sequences, within ID mean ± sd = 0.33 ± 0.15, between ID = 0.29 ± 0.13, adjusted *p* = 1). Lastly, when comparing within-individual LSI scores between the two sexes, adult females showed significantly higher LSI scores compared to males (Mann-Whitney *U* = 30,548, *N* = 60 individuals, *n* = 162 sequences, adjusted *p* < 0.001; Fig. 4) while there was no difference between the sexes for subadults (Mann-Whitney *U* = 27,646, *N* = 39 individuals, *n* = 129 sequences, adjusted *p* = 1; Fig. 4).

## Discussion

The investigation of the call order and combinatorial structure of the six sentinel call types occurring in meerkat sentinel sequences showed that the different sentinel call types were produced in a graded way, demonstrating the following gradation pattern: single-note, double-note, triple-note, multiple-note, di-drrr, wheek and alarm calls. Call repetitions within the same call type were highly over-represented, while transitions between call types mostly occurred within one step up or down the gradation pattern. Analysing sequence similarity within and between individuals and groups demonstrated that the short-note calls, but not sentinel warning or alarm calls, displayed high within-individual consistency, whereby adults and females had higher consistency scores than subadults and males respectively.

In previous work, the perceived predation risk was experimentally manipulated by playing back alarm calls to sentinels, thereby simulating that another group member has spotted a predator close by, and thus increased a sentinel’s perceived risk [23]. The results demonstrated that single- and double-note calls (i.e. calming calls) were produced when the perceived risk was low (before the alarm playbacks and after visual scanning of the surrounding area by the sentinel to confirm that no predator was in sight), while di-drrr and wheek calls (i.e. warning calls) were produced when perceived risk was high (immediately after the playbacks of alarm calls). Taking these results together with the gradation found in this study, we argue that it is very likely that the order of sentinel call types within sentinel sequences over longer time periods is graded and correlates with the caller’s perceived predation risk.

By comparing the different diagonals of the observed transition matrix to randomly generated sequences, we found that besides repetition of the same call type, individuals are more likely to move up and down the gradient, confirming the a priori assumed gradation pattern from sentinel calming calls to triple and multiple notes, to the two types of sentinel warning calls and lastly to alarm calls. Repetitions of the same call type were much more frequent than expected by randomisations and most transitions between call types were one-step changes up or down the risk level (i.e. the 1 and − 1 diagonals are highly over-represented, while the 2 and − 2, as well as the 3 and − 3 diagonals were significantly less frequent than expected). For the short-note calls, this is in line with a large body of literature where the temporal structure of the same call element, here the single-note calls, varies with increasing risk or arousal state [25–27]. However, here we provide evidence for a novel pattern of gradation including different call types—the short notes, the sentinel warning calls and alarm calls—which differ substantially in their acoustic structure and while the transition from sentinel warning calls to alarm calls may be more gradual, no intermediate calls between the two sentinel call types have been observed. Therefore, these vocal sequences present a gradation over multiple, structurally distinct but functionally related call types, which very likely conveys information about the immediate perceived predation risk.

The difference in the 5th diagonal, which was under-represented when risk was increasing (diagonal − 5) and as frequent as expected when risk was decreasing (diagonal 5), provides some indication that with increasing perceived risk the gradation of call types might be more conservative than with a decrease in perceived risk. Meerkats continually scan the area for the presence of predators and often spot potential threats from long distances ([22]; personal observation MM and RR), when it is not clear yet whether the spotted object presents a threat or not. Thus, rather than getting surprised by any potential threats and make larger steps up, they keep looking at it and as it gets closer (personal observation MM and RR), and thus may gradually move up the gradation pattern one level at the time. Once the threat is close enough for identification, sentinels either produce the appropriate, functionally referential alarm call, or—if it turns out to be non-dangerous—they seem to directly drop in gradation to the all-clear calls. Therefore, sentinel call sequences produced during decreasing perceived risk might be less strictly graded than during increasing risk, as it seems more efficient to directly give the all-clear sentinel calming calls when the potential threat turns out to be non-dangerous.

The higher sequence consistencies we found within individuals compared to between individuals were mostly based on the four categories of short-note calls, as taking only warning and alarm calls into account did not show any individual consistency. This could indicate that short-note calls might relate to the internal state of the caller, resulting in individually distinct calling patterns, while warning and alarm calls might be produced in response to external stimuli, therefore not showing any consistent individual differences. The exact mechanism of the individual patterns, however, remains unclear. There are several possibilities how sequences can be individually distinct, while still following the overall observed, risk-related gradation pattern. First, the over-representation of repetitions of the same call type could allow for individual-specific repetition patterns before transitioning to the next call type. Secondly, consistent differences in arousal state between individuals (reviewed in [28, 29]), which may reflect differences in personality [30, 31], could result in some individuals moving up or down the risk level much quicker than others. Closely related to this, it is possible that although all individuals produce all six types of sentinel calls, the rate of using specific call types may differ between different individuals (e.g. some individuals may use more often triple-note calls and others more double-note calls). Further research is needed to investigate the exact mechanism and composition of individually distinct sentinel sequence patterns.

Regardless of the exact mechanism underlying the individual variation in sentinel sequences, consistent call patterns could convey relevant information about the individual identity of the caller. From a signalling perspective, encoding individual identity through call patterns, as well as frequency and temporal-based acoustic parameters of single calls, represents redundant information, i.e. the same information is encoded in different ways [32, 33]. Redundancy is a common feature in animal vocal communication [34] and is thought to increase the ability of the receiver to correctly detect the relevant information of a vocal signal and thus provides signal robustness, especially in noisy environments [35]. In the context of sentinel behaviour only alarm calls directly refer to a spotted predator, while the sentinel calls refer to a lower perceived risk, which is likely to be differently assessed by different individuals. Thus, having redundant information about the individual on sentinel guard—especially in open and sometimes very windy habitats where call propagation might be impaired at times—could be an adaptive strategy to facilitate receivers’ assessment of the provided social information [36]. This would be in line with previous work demonstrating the high importance of individual identity in use of social information provided by the sentinels [24]. However, it is yet to be tested experimentally if the found temporal structures are meaningful for the receivers of the signal.

The higher sequence similarity in females compared to males may be related to the sex differences in dispersal. Females stay in their natal group, potentially experiencing the more stable social environment, while males disperse from their natal group and either join another group or find a new group. Further research focusing on males before and after dispersal could improve our understanding of whether individual-specific sentinel sequence patterns are consistent within adults over time, or whether they develop, and if so, how changing groups affects individual calling patterns. The lower within-individual consistencies of sentinel sequences in subadults indicate that the individual-specific call pattern produced by adults is not yet fully developed when young meerkats start to go on guard, but rather undergoes ontogenetic development. Thus, ontogenetic changes of sentinel calls may not only be related to the honing of skills and increased certainty in assessing the immediate risk (Rauber & Manser, in prep.), but also to the development of individual-specific call patterns compared to other litter members or the rest of the group.

## Conclusions

This study supports previous work that not only single acoustic units contain meaningful information, but the order of acoustically different single units in longer sequences can convey additional information. Moreover, our work highlights the large compositional variety we find in animal vocal sequences, by demonstrating that sequences can contain multiple levels of temporal structuring related to multiple functions. We present a novel type of combinatoriality underlying animal vocal sequences, which neither fits what has been described for animal songs nor the typical combinations of meaningful units described so far. We demonstrate that by combining different call types in a graded way into one sequence, meerkats convey meaningful information about subtle changes in the external environment, while at the same time the temporal pattern of the distinct calls contains stable information about caller identity. Similar mechanisms may underlie temporal variation in animal songs, combining information on the arousal of the caller based on physiological and environmental changes as well as on individuality. Our work emphasises how seemingly complex call sequences can be described by simple rules, in this case gradation across distinct call types related to contextual characteristics, combined with individual-specific call patterns. Understanding the underlying mechanism and information content of animal vocal sequences ultimately improves our knowledge about the evolution of combinatoriality in animal communication systems and potentially our own language, where combinatoriality plays a major role in the generative production of meaningful information [2].

## Methods

### Study site and species

Data were collected at the Kalahari Meerkat Project (KMP) located at the Kuruman River Reserve in the southern Kalahari Desert, Northern Cape, South Africa (for more information about habitat and climate at the study site, see [37, 38]). All group members were uniquely dye marked to allow individual identification, and one or two individuals of each group were fitted with a radio-collar to facilitate localisation of the group [39]. All groups were habituated to close human observations, allowing us to perform high-quality recordings within a distance of 0.5 m to the focal individual.

### Sound recordings

Sound recordings of individuals on sentinel guard were collected during May–December 2014, January–July 2016 and February–July 2017. Calls from naturally occurring sentinel events were recorded using a Sennheiser directional microphone (ME66/K6) connected to a Marantz PMD-670 solid-state recorder (Marantz Japan Inc.; sampling frequency 44.2 kHz, 16 bits accuracy). A windshield (Rainhardt, W200) was attached to the microphone to ensure high-quality recordings under variable wind conditions. The microphone was fixed to a 1.5-m-long telescopic pole in order to maintain a recording distance of about 0.5 m and a high signal-to-background ratio. In total, we collected 221 recordings from 73 adult sentinel individuals from 15 different groups, as well as 193 recordings from 51 subadults from 10 different groups. One sequence of sentinel calls consisted of all calls produced during one whole sentinel period, which equals one recording event with a median duration of 3.27 min (range = 0.18–28.68 min). To avoid any bias due to very short recordings, we removed all recordings with less than 10 calls resulting in 162 recordings from 60 adults (median 4 recordings per individual, range 1–10) and 129 recordings from 39 subadult individuals (median 5 recordings per individual, range 1–8). Although some individuals were only recorded once or a few times, they were still included in the analysis as it has been demonstrated that these data points are still valuable to assess between-individual variation [40].

### Sound analysis

All vocalisations contained in the collected sound recordings were manually assigned to one of the six described sentinel call types and alarm calls (Fig. 1; see Additional file 2: Fig. S2 for five 30 call type long sub-sequences of example sequences) using a combination of visual and acoustic inspection of the spectrogram in Adobe Audition (2015.0 Release) [22, 23]. Work on previously described meerkat call combinations found that the silence interval between two combined calls is generally 20 times less than the silence interval among vocalisations that were considered to be independently produced [16]. We used the same criteria here to categorise each of the six types of sentinel calls [22] (see Additional file 3: Fig. S3 for a 15 second cut-out of a sequence containing sentinel calls and the respective silence intervals within and between call types). After the call categorisation, we recorded the temporal order of each call within a sequence for each recorded sequence, as well as a caller’s identity, its group affiliation, the group it was born in (natal group), age and sex.

### Constructing a transition matrix

To analyse the temporal order of call types produced in sentinel sequences, we constructed a transition matrix containing the number of transitions from each sentinel call type to any other sentinel call type. Details of how to construct a transition matrix have been described in [41]. To summarise, the resulting transition matrix containing all transitions from all recordings contains seven rows and seven columns (six sentinel call types plus any type of alarm calls pooled together into an alarm call group). Each cell, for example row sn and column dn, is filled with the count of the number of times a meerkat has transitioned from the column call type (e.g. sn) to the row call type (e.g. dn). The diagonal cells (zero diagonal) represent repetitions of the same call type. Based on these counts, we calculated the transition probabilities as the count of each cell divided by the sum of the row. Accordingly, the transition probability describes the probability a specific call type is given conditioned on the preceding call type.

### Testing gradation of sentinel call types

To test if the order of sentinel call types is graded, i.e. whether the six sentinel call types and alarm calls are produced in a graded, stereotypic order, we randomised the call sequences within each recording of a sentinel period 1000 times. By randomising within a recording, we kept the overall frequencies of the calls constant while randomising the call order. We then calculated the sums of each diagonal, expecting that if a call is given in a highly graded way, the likelihood of a call transition should be getting smaller further away from the zero diagonal (see Additional flie 1: Fig. S1). For example, individuals that just produced a triple-note call are expected to either stay on the triple-note calls, go one level down to the double-note calls or go one level up to the multiple-note calls. To test this, we calculated the 95% confidence intervals for each of the diagonals from the 1000 randomisations and compared it to the value of the observed transition matrix across all recordings.

As self-replications of the same call type were highly over-represented in our call sequences, we then did a second round of randomisations where we focused only on call transitions between call types. To do this, we kept the number of repetitions of the same call type (zero diagonal) constant and randomised all other transitions which occur between two different call types. Again, we then calculated the diagonal sums and their 95% confidence intervals and compared them to the values from the observed transition matrix.

All correlations between call gradation and the sentinel’s perceived risk are based on a previous study where predation risk was experimentally manipulated by playing back alarm calls to sentinels [23].

### Comparing similarity between sequences

Recent advances in the analysis of acoustic sequences have introduced the Levenshtein distance (LD) as a robust analytical tool to compare animal vocalisations [42, 43]. The LD is a pairwise comparison of two sequences of potentially different length that, after prior alignment, calculates the minimum number of point changes—insertions, deletions or substitutions—to get from sequence A to sequence B [42–44]. We then calculated the Levenshtein Similarity Index (LSI) for each of the pairwise comparisons of two sequences. The LSI score takes the length of the longest sequence into account and thus how many potential point changes (number of insertions, deletions or substitutions) are possible, therefore controlling for the fact that longer sequences have a higher probability of containing more differences than smaller sequences. The LSI score is calculated as 1 − LD/max length of sequence. The resulting scores vary between 0 and 1 whereby 1 indicates complete similarity and 0 indicates complete dissimilarity between the two tested sequences. To investigate consistency of sequences within recordings, we divided recordings into three parts and calculated the LSI using the first and the last third of the sentinel recording. To test if there were any additional group, natal group or individual signatures contained in sentinel sequences, we compared recordings from within and between groups (i.e. group signatures), as well as whether individuals originated from the same or different natal groups (to take into account that this signal could emerge early in the vocal development and therefore resemble the call patterns present in the group they were born in) and lastly within and between individuals to test for individual-specific call patterns. To specify which calls within the sequences contributed most to the observed individual patterns, we compared the LSI within and between individuals using first all six sentinel call types, then using only short-note calls (which add up to more than 95% of all sentinel calls produced [22]) and lastly replacing all short-note calls between di-drrr and wheek calls with a single X and therefore only taking the sentinel warning calls into account while keeping their relative distances constant (i.e. whether they are produced adjacent or not).

### Statistical analysis

All statistical analyses were done using R version 3.5.2 [45]. To test the collected sequences for group-, natal group- or individual-, age- and sex-specific signatures, we calculated the LSI using the packages *stringdist* [46] and *RecordLinkage* [47]. We then conducted a non-parametric Mann-Whitney/Wilcoxon test to compare the LSI scores within and between groups, within and between natal groups respectively as well as within and between individuals. For the latter, we furthermore compared within-individual LSI scores between males and females as well as subadults and adults. We used Bonferroni-Dunn correction to adjust the *p* values to control for multiple pairwise comparisons.

## Supplementary information

**Additional file 1:** Fig. S1. The different diagonals of the constructed transition matrix, including call transitions between single note (sn), double note (dn), triple note (tn), multiple note (mn), dir-drr (didr), wheek (wh) and alarm calls (al). The central, zero diagonal (dark grey) represents the repetitions of the same call type, first order diagonal (light grey) indicate call transitions change one step up or down in the expected gradation hierarchy and so forth. In a graded system, the diagonals closer to the zero-diagonal are expected to be overrepresented, while the diagonals further away are expected to occur less frequently than by chance.

**Additional file 2:** Fig. S2. Five 30 call type long cut-outs of examples sequences produced by five different sentinels (A-E).

**Additional file 3:** Fig. S3. Spectrogram showing a 15 second cut-out of an example sequence consisting of single note and double note calls with silence intervals between them.

## Data Availability

The datasets generated and analysed during the current study are available from the corresponding author on reasonable request.
