# Supplementary material for: Call order within vocal sequences of meerkats contains temporary contextual and individual information
Source: BMC Biol. 2020 Sep 9;18:119. doi: 10.1186/s12915-020-00847-8 (PMC7488032; doi:10.1186/s12915-020-00847-8)
Supplement: Supplementary file 1 — Additional file 1: Fig. S1. The different diagonals of the constructed transition matrix, including call transitions between single note (sn), double note (dn), triple note (tn), multiple note (mn), dir-drr (didr), wheek (wh) and alarm calls (al). The central, zero diagonal (dark grey) represents the repetitions of the same call type, first order diagonal (light grey) indicate call transitions change one step up or down in the expected gradation hierarchy and so forth. In a graded system, the diagonals closer to the zero-diagonal are expected to be overrepresented, while the diagonals further away are expected to occur less frequently than by chance. [file 12915_2020_847_MOESM1_ESM.docx]

**Additional file 1: Fig S1.** The different diagonals of the constructed transition matrix, including call transitions between single note (sn), double note (dn), triple note (tn), multiple note (mn), dir-drr (didr), wheek (wh) and alarm calls (al). The central, zero diagonal (dark gray) represents the repetitions of the same call type, first order diagonal (light gray) indicate call transitions change one step up or down in the expected gradation hierarchy and so forth. In a graded system, the diagonals closer to the zero-diagonal are expected to be overrepresented, while the diagonals further away are expected to occur less frequently than by chance.
