# Supplementary material for: Call order within vocal sequences of meerkats contains temporary contextual and individual information
Source: BMC Biol. 2020 Sep 9;18:119. doi: 10.1186/s12915-020-00847-8 (PMC7488032; doi:10.1186/s12915-020-00847-8)
Supplement: Supplementary file 2 — Additional file 2: Fig. S2. Five 30 call type long cut-outs of examples sequences produced by five different sentinels (A-E). [file 12915_2020_847_MOESM2_ESM.docx]

**Additional file 2: Fig. S2.** Five 30 call type long cut-outs of examples sequences produced by five different sentinels (A-E)
