# Supplementary material for: Call order within vocal sequences of meerkats contains temporary contextual and individual information
Source: BMC Biol. 2020 Sep 9;18:119. doi: 10.1186/s12915-020-00847-8 (PMC7488032; doi:10.1186/s12915-020-00847-8)
Supplement: Supplementary file 3 — Additional file 3: Fig. S3. Spectrogram showing a 15 second cut-out of an example sequence consisting of single note and double note calls with silence intervals between them. [file 12915_2020_847_MOESM3_ESM.docx]

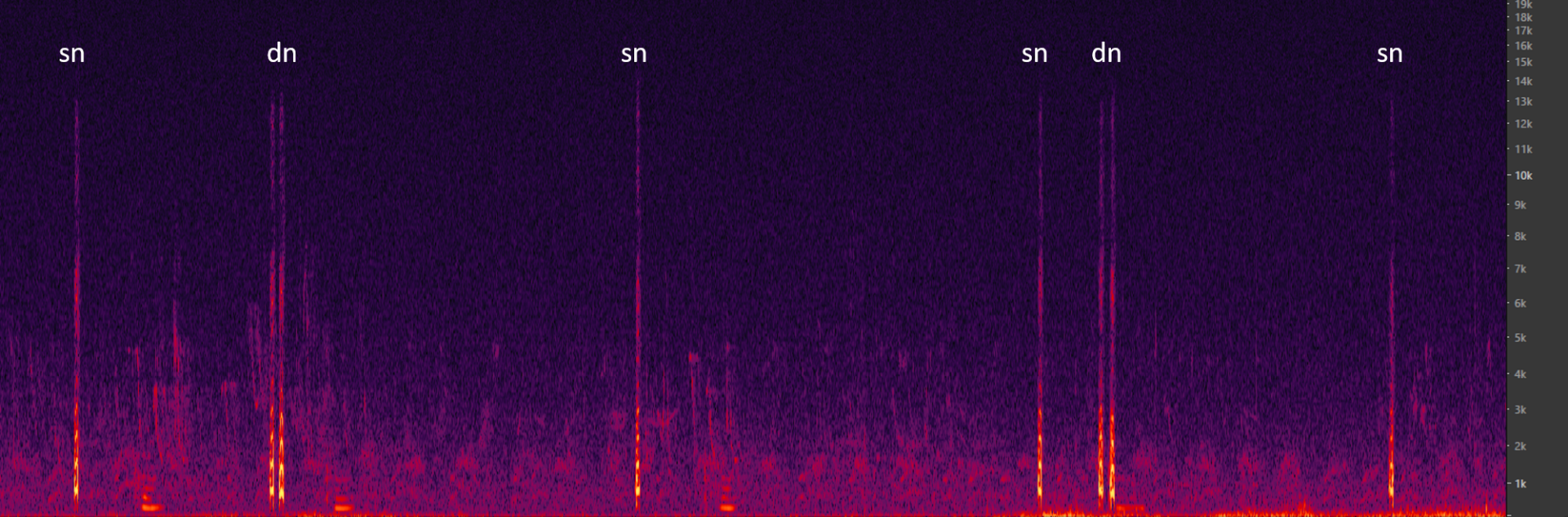


**Additional file 3: Fig. S3:** Spectrogram showing a 15 seconds cut-out of an example sequence consisting of single note and double note calls with silence intervals between them.
